# Completing upper secondary school: Results from a ten-year longitudinal study in Norway

**DOI:** 10.1371/journal.pone.0361808

**Published:** 2026-11-06

**Authors:** Rannveig Grøm Sæle, Tore Sørlie, Charlotte Bjørnskov Goll, Oddgeir Friborg

**Affiliations:** 1 Department of Psychology, UiT The Arctic University of Norway, Tromsø, Norway; 2 Department of Clinical Medicine, UiT The Arctic University of Norway, Tromsø, Norway; 3 Department of Mental Health and Substance Abuse, University Hospital of North Norway (UNN), Tromsø, Norway; University of Granada: Universidad de Granada, SPAIN

## Abstract

The completion of education is important both for the individual and society. High dropout rates from upper secondary school have been a national problem in Norway, and especially in northern Norway. Academic aptitude or grades (GPA) are important, but given the multifaceted nature of dropout, we conducted a longitudinal analysis to examine predictors of school completion after ten years. The study included 1676 students in Troms County (latitude 69 degrees north) starting in upper secondary education in 2010. We included a broad set of predictors related to school performance, psychological factors such as vulnerability, resilience, personality, and mental health, alongside demographic data. The mixture modeling revealed three completion trajectories: normal progression (40.1%), delayed completion (36.6%), and non-completion (23.3%). Correlation analyses showed significant relationships between completion and several of these predictors; however, in multinomial regression analyses, no variables beyond GPA significantly predicted the normal progression trajectory, whereas delayed completion was additionally accounted for by study track choice and self-perceived health, with GPA playing an even stronger role. This underscores the importance of prior school performance, but we also discuss the implications of the findings for the delayed-completion group.

## Introduction

A substantial number of studies have shown favorable benefits of completing education, such as better mental and physical health and quality of life [1,2], higher employment rates and income levels, more engagement in society, less crime [3–5], and reduced mortality [6]. On a societal level, an educated population also implies less money spent on social welfare, disability pensions, health care and justice/prisons services. For the individual, education offers better opportunities for social mobility [7].

In Norway, almost all adolescents move on to upper secondary schooling after finishing lower secondary. Following the national education policy reform in 1994, which legally ensured all adolescents the right to continue upper secondary schooling, few students choose otherwise despite it not being mandatory [8]. Dropout has on the other hand become a pronounced problem. In 2010, only 71% of Norwegian students had completed upper secondary within five years after they started, compared to the OECD average of 81% [9]. The number of completers was consistently worse in the northern counties as compared to southern Norway, being 64.3%, 62.3% and 51.4% in Nordland, Troms and Finnmark, respectively [10]. Although today’s number of students completing the entire upper secondary program has improved noticeably, about 18% still do not complete school within five/six years [11].

These numbers motivated a longitudinal study in Troms County in 2010 to learn more about the reasons for dropping out of secondary schooling. At the time, few studies had explored underpinning reasons for quitting prematurely. Most studies included a narrow range of potential predictors, a pattern that persists today, constraining the conceptual range, which is unfortunate given the multifaceted nature of the dropout phenomenon. The present study, therefore, assembled a comprehensive survey including a broad set of potential explanatory variables and secured access to registry data to obtain objective information on school completion status for the entire sample. The knowledge background in 2010 led us to put a survey registering information about demographic, school-related, motivational and psychological variables relevant for completing upper secondary.

### Demographic and school-related predictors for school completion

The role of gender for completion varies across studies internationally [12], however in Norway, the largest share of dropouts are among boys. Students in the vocational track also drop out more often than those in the general track [11]. Students with parents having upper secondary or higher education are more likely to complete than their peers with parents without education.

Among school-related variables, previous grades or other measures of academic achievement are one of the single-most important predictors for completing an education across countries, cultures, or study program levels [13,14]. In Norway, almost all students (98.8%) starting in 2016 with more than 50 grade points from lower secondary school (range: 10–60), completed within 5 years, and almost all of them also within the normed time of 3 years [11]. Among students with 10–29 grade points, the rate was 44.1%, of which only 25.9% completed within the expected standard time. Exam grade points, even after adjusting for other predictors, remain the primary explanatory variable and should therefore be included in any dropout analysis. Previous analyses of the current sample have already shown that grades are most important [15,16], but since the present study had access to ten-year follow-up data, they are reanalyzed.

### Psychological predictors of school completion

Psychological predictors typically include mental health status, as well as students’ self-evaluations of motivation, scholastic and personally characteristics, and exposure to adverse life events [14]. Studies examining the relationship between stressful life events (SLE) and somatic or mental health show a consistent negative association, albeit weak in magnitude (e.g., [17,18]). Because poorer health is generally expected to affect attention, energy levels, and cognitive capacity for studying, SLEs may contribute negatively to dropout, particularly for adverse events.

Adequate to good adaptation following adversity is often characterized as resilience [19], which may be augmented by intrapersonal protective factors like cognitive skills, personal and social competencies, or by interpersonal factors like family cohesion, social resources or other support systems that reinforce adaptation. A previous study showed a somewhat unexpected negative association between higher social competence and grades that may be explained by highly social students spending more time with friends at the cost of time for homework [15]. Social competence may nevertheless offer protection against dropout through the elicitation of positive motivational responses from others [20]. Sense of coherence (SOC) is a related construct explicitly linked with the salutogenic model of what facilitates health and well-being despite adversity. According to Antonovsky [21] it represents a global, pervasive and dynamic orientation towards life that increases (1) comprehensibility, by making internal and external stimuli stand out as structured, predictable and explicable, (2) manageability, by having access to the necessary internal or external resources to meet life demands, and (3) meaningfulness, which motivates the desire to find resolutions to life challenges [22]. The development of SOC is assumed to be continuous from birth to early adulthood (age of 30), and to foster problem-solving of life demands and thus health promotion [23]. Winding and colleagues [14] found that low SOC at the age of 14–15 predicted later dropout from vocational education, but few other studies have examined this relationship.

The role of mental health for school completion is a debated issue as the level of depression, anxiety, and worry during this period of adolescence increases. Specifically, stress and pressures related to school work are highly frequent, as approximately every other student reported it in a national Ungdata survey; however, most adolescents do not report it as problematic [24–26]. A potential connection between mental health and educational achievement may run both ways, as dropout might do as well [27]. Adolescent mental health may be of an internalizing domain (e.g., depression, anxiety), but also an externalizing domain (e.g., hyperactivity, behaviour disturbances). Both domains have shown a negative association with academic performance and dropout [28].

The current study thus explored relationships between these sets of variables and completion status to examine which best predicted completion ten years after beginning upper secondary schooling.

*Hypothesis:* We hypothesize that higher previous grades will be the strongest predictor of completing upper secondary. In addition, we hypothesize that some of the previously less investigated variables, like sense of coherence, resilience, and the masculine/feminine measure of personality, would contribute to explained variance in completion, whereas the remaining predictors are less clear.

## Materials and methods

The current study was conducted in Northern Norway. After 10 years of mandatory schooling, Norwegian students have a legal right to a free place in upper secondary education. They can choose from different programs within the larger categories of either the general track (qualifying for higher education) or the vocational track (qualifying for a practical occupation). The general track has a normed duration of three years, while vocational track normally takes four years, comprised of two years of school-based education followed by a two-year apprenticeship. Students may switch between programs, and those on a vocational track may also complete an extra year of general schooling to qualify for higher education.

### Participants

All students entering upper secondary school in Troms County in the fall of 2010 were invited to participate in the study. Data collection took place from 31.08.2010–20.09.2010. Of 2434 eligible students, 1676 consented (response rate = 69%). The mean age was 16.7 (*SD* = 1.3, 48% females). The proportion of general and vocational track students were 46% and 54%, respectively. The gender and study track proportions corresponded to the pupil population proportions at the time of data collection [10].

### Design and procedure

The study design was longitudinal, with the first wave of questionnaire data collected during fall 2010, the second during spring 2013 (T2; *N* = 664) and the third during spring 2020 (T3; *N* = 534). Because of the low response rates at T2 and T3, and with only 242 participants participating at all three waves, only the baseline self-report data are included in the present analyses. The registry data on completion status were obtained from the Troms County authorities.

### Ethics

The students received written and oral information about the study at school by project personnel. Consenting students completed a questionnaire during a classroom session. Parental consent was collected from participants below the age of 16. The study was approved by the Regional Committee for Medical and Health Research Northern Norway (REK North; reference number 2010/1503).

### Measures

*Grades* from lower secondary education were obtained from Troms County. There are missing data for 19.3% of cases (some students had grade exemptions for different reasons, and some students had a Steiner school background where grades are not given). Total range for GPA in Norway is 1–6, in this sample values ranged from 1.56-5.94.

*Socio-economic status (SES).* Unfortunately, a large share of adolescents did not know their parents’ educational level and responded ‘don’t know’ on a question addressing this. Hence, we chose another aspect of socio-economic status, family income level. This was measured by a relative question asking the pupil to compare their family’s financial situation with their perception of other families’ economic situation using four categories: 1-badly off, 2-medium of, 3-well off and 4-very well off.

We also included a question about educational plans for the future, as well as a few items regarding different aspects of school life: “When lessons are over and assignments are given, to what degree are you capable of solving these tasks on your own?”; “In the classroom, there are certain rules about what is allowed during lessons. To what extent have you come into conflict with these rules?”; “I am bullied or harassed at my school”. These three items were answered on a six-point Likert scale from 1) to a little degree, to 6) to a large degree.

*Literacy problems* were measured by an eight-item scale addressing difficulties with decoding, reading comprehension, spelling, and written expression. Sample items are “Do you have reading difficulties?” and “Do you make many writing mistakes?”, and participants answered yes or no. The scale has shown satisfactory psychometric properties [15]. Cronbach’s alpha was  .83.

*General health* was measured by one item asking simply: “How do you rate your overall health?” The response categories were 1) very good, 2) good, 3) not very good and 4) poor.

*Mental health* was measured by HSCL-5, which is a five-item version of HSCL-25 (Hopkins Symptom Checklist). It measures internalized mental problems during the past two weeks with four response categories: 1) not at all bothered, 2) a little bothered, 3) quite, and 4) very much. Internal consistency was good (Cronbach‘s α = .85), and the short version is previously found to be comparable to the full version [29].

*Satisfaction with life* scale [30] consists of five items answered on a Likert scale ranging from 1) perfectly true to 7) not true. Sample item: “I am satisfied with my life”. Higher scores indicate poorer satisfaction with life (Cronbach’s α = .89).

*Sense of coherence* scale [22], comprises 13 items asking questions about meaningfulness, comprehensibility and manageability. Response format is a 7-point Likert scale. One sample item is “Until now your life has had…” with response options: “No clear goal or purpose at all” [1] to “Very clear goals and purpose” [7]. Scores were reversed before analyses so that higher scores indicate poorer sense of coherence (Cronbach’s α = .84).

*Resilience* was measured by a short version of the 33-item Resilience Scale for Adults (RSA; [31]). Twelve items were used to assess four factors: positive perception of self, social competence, family cohesion, and social resources (Cronbach’s α = .76,  .88,  .86, and  .89, respectively). Sample item is “I find new friends easily”. Response format ranges from 1) totally agree to 5) totally disagree.

*Social support* was assessed by six items rated on a 1–5 scale. These were “Do you feel that you belong to a community with people who trust each other and commit to each other”, “How often do you talk to your friends”, “Do you have anyone near you who is considerate, attentive, and interested”, “Do you get necessary help and support from family and friends if you are ill”, “Do you have friends outside of your family appreciating you”, and “Do you ever feel lonely” (reversed item). (Cronbach’s α = 0.69).

*Motivational focus* was measured by the General Regulatory Focus Measure [32], addressing the degree the respondent agrees with either a promotion or prevention motivational focus [33]. A promotion focus is driven by the pursuit of positive outcomes, whereas a prevention focus is driven by the avoidance of negative outcomes. Sample items are “I typically focus on the success I hope to achieve in the future” (promotion) and “I frequently think about how I can prevent failures in my life” (prevention). Cronbach’s α was 0.88 for promotion and 0.79 for prevention.

*Personality traits* were measured by the Personal Attributes Questionnaire [34]. This questionnaire addresses personality traits typically associated with gender and consists of three subscales. The masculine and feminine subscales include traits seen as socially desirable in both genders but that are more prevalent in either men (e.g., independent) or women (e.g., understanding of others). The masculine/feminine subscale includes traits supposedly seen as favorable in one gender but not in the other (e.g., aggressive, home-oriented). Cronbach’s α was  .73 for the masculine subscale,  .82 for the feminine subscale, and  .46 for the masculine/feminine subscale. The latter was hence excluded from further analyses due to the low internal reliability.

*Stressful life events* were indexed by asking whether specific events had occurred in their life, for instance, serious illnesses, threats of violence or sexual assaults.

*Total sleep time* (in hours) was calculated as the time period from going to bed and waking-up during schooldays, minus the sleep onset latency, as according to the Munich ChronoType Questionnaire [35](MCTQ; Roenneberg et al., 2003).

### Outcome variable

Registry data on completion status were available from Troms County for all years until 2020, except for 2016. Because of a temporary administrative merger between two counties in Northern Norway in 2015−16, the counties’ databases were combined, thereby precluding county-specific separation. This represented a minor restriction given that the main outcome was the students’ final status in 2020. The completion variable was coded into four groups, of which only the two first were used as outcomes in the analyses: 1) dropout (including students without grounds for a diploma, for instance because of too much absence, and students not registered anywhere in the county), 2) completion and pass, 3) completion, but not pass (e.g., with failing grades) and 4) still in school. To compare our completion rate with the official statistics, we use data from five years after enrollment. Statistics Norway reports that 67% of the 2010 cohort had completed upper secondary in Troms County in 2015 [36], while the number was 76.5% in our sample.

### Statistical analyses

We fitted a longitudinal latent class mixture model to repeated annual binary completion data (0-not completed, 1-completed) using Mplus version 8.9 [37]. The Bayesian Information Criterion (BIC) and the Bootstrapped Likelihood Ratio Test (BLRT) were used to decide the number of latent classes, or trajectories. Lower BIC values and/or a significant BLRT after adding a class support this; however, we did not retain an extra class if the BIC reduction stopped or worsened and the BLRT was non-significant. Entropy is reported to indicate classification certainty (0-no separation, 1-perfect, > 0.8 good, > 0.9 excellent separation). A key reason for the latent class approach is that the observed completion indicator was reversible (not necessarily monotonic), so students might complete the curriculum after 3 years but then switch to another curriculum that they did or did not complete. An observation-based classification based on the first occurrence could thus prematurely assume a completion status, whereas a model-based classification would use probability estimates based on the entire series of completion data to characterize the trajectory. The latent thresholds were estimated as free within and equal between classes, whereas the factor means (i.e., propensity of completion) were free, allowing separation of the classes by event timing and level (i.e., probability for completion). The latent classes thus represent empirically distinct longitudinal trajectories under a common measurement structure. The analysis produces posterior probabilities of latent class membership for each student and assigns each student to the class with the highest probability, which makes the decision probabilistic rather than deterministic. The trajectories thus provide a parsimonious representation of the heterogeneous completion patterns observed in the data, and characterize students who share common longitudinal features in how they progress through the educational system. The categorical latent class variable represents a longitudinal characterization of completion pathways that we regressed on covariates within a multinomial regression framework. Given the very high entropy, as in these models, classification certainty was very high, and we saved and exported the latent classes to IBM SPSS Statistics 29 [38]. We regressed the predictors on the latent classification variable in two steps: 1) all demographic, school-related, and psychological variables, and 2) the grade point average (GPA) variable, together with significant variables (*p* < .05) from the previous step. To maximize the available sample size, we did not include variables with substantial missingness in the final multivariable model unless preceding analyses showed evidence of association with trajectory membership. Multicollinearity was of less concern as VIF ranged from 1.1 to 2.4. An inspection of Cook’s distance revealed 10 and 5 cases for the two multinomial regression analyses, respectively. These were removed to examine the sensitivity of the findings.

We performed an attrition analysis comparing baseline (T1) characteristics between participants who remained in T2, T3, or both, with those who only responded to the baseline survey. Retention was significantly associated with higher GPA (Cohen’s *d* = 0.42 at T2; 0.35 at T3), female gender (χ^2^(1) = 59.9 at T2; 19.4 at T3, both *p* < .001) and general track (χ^2^(1) = 10.7, *p* = .001 at T2; 7.67, *p* = .006 at T3), and to a smaller extent, less literacy problems, higher task solving ability, less conflict with school rules, and higher perceived social support (all *d*s ≤ .29). Attrition was thus not completely at random, but strongly linked to better academic performance, gender, track and general functioning.

## Results

The proportion of students completing upper secondary after ten years was 77.6% (spring 2020), whereas 19.7% dropped out or were not registered, 2.7% completed without a passing diploma, and 0.5% were still in school.

Descriptive statistics and correlation coefficients between all variables are available in S1 and S2 Tables. Both tables include completion status and GPA information, which were significantly correlated; that is, a higher GPA was positively associated with completion. As shown in S2 Table, significant demographic coefficients, in descending order of strength, were educational track, literacy problems, educational plans, gender, and task-solving abilities (all *p*’s < .001). Of the psychological variables, general health, social support and masculine personality traits were significant (*p*’s < .001). These associations were in the expected directions and indicate that school-related and psychological variables are associated with school completion, but to a lesser extent than GPA. Furthermore, GPA also correlated significantly with other demographic, school-related and psychological variables, indicating its broader importance for general health, functioning, and well-being.

### Latent completion trajectories

The discrete-time mixture analysis showed best model fit for extracting three latent trajectories (BIC = 4806.6), while two or four trajectories had worse BIC (5900.4 and 4821.5, respectively. The BLRT supported this decision, as it was significant for one vs. and two and two vs. three latent trajectories (both *p*’s < .001), but not three vs. four. The entropy was high (0.975), indicating very high classification certainty. See Fig 1 for a visualization of the completion process over a 10-year period for the three student groups. Years 8 and 9 had to be excluded in the estimation to avoid a non-positive definite product matrix warning.

**Fig 1:**
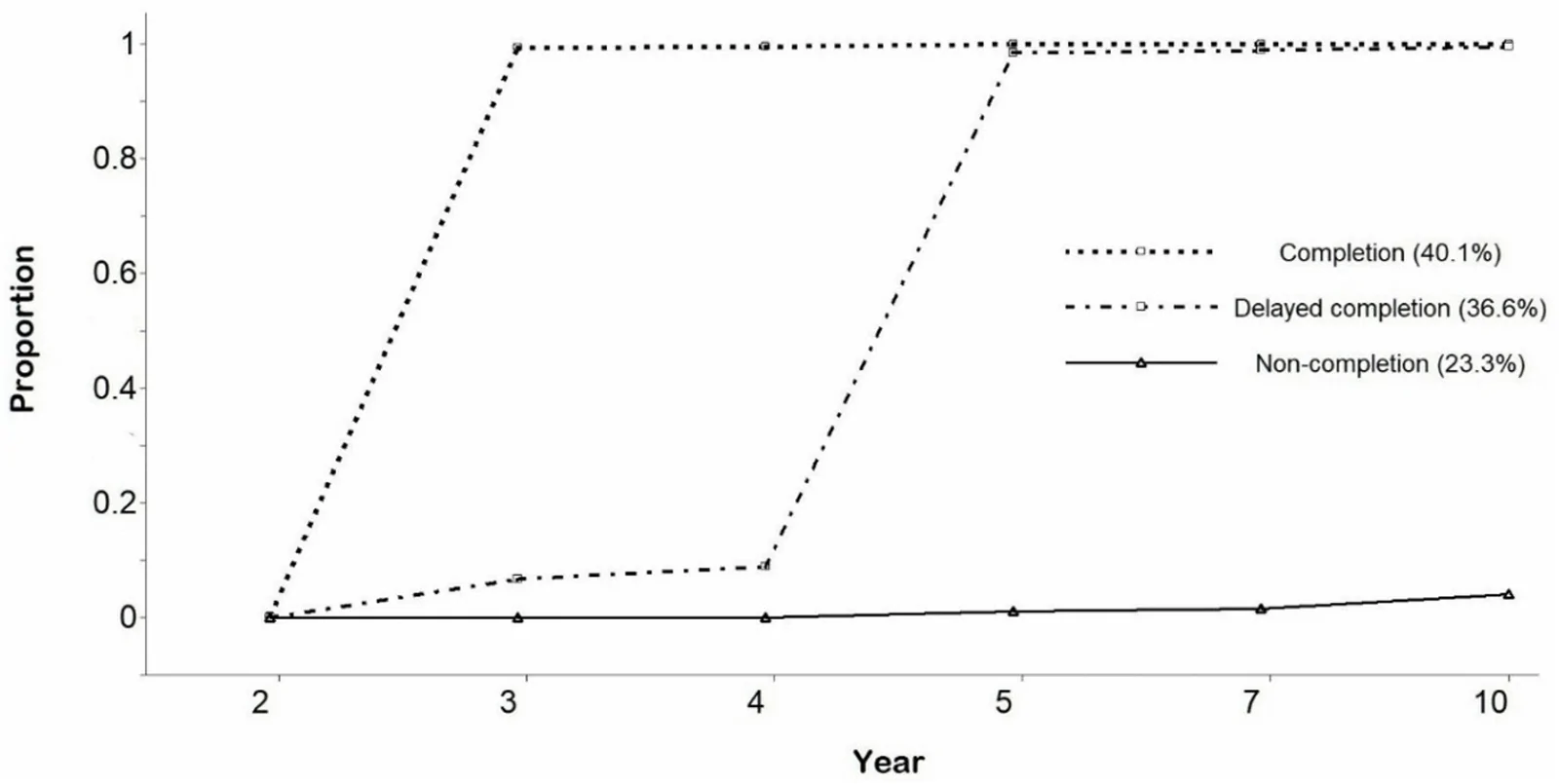
Latent completion trajectories.

Table 1 shows all 11 observed completion patterns as represented by the three latent trajectories. Numbers in the completion sequence column describe non-completion (0) versus completion (1) status for each year from 3 to 10.

**Table 1:** All 11 Observed Completion Patterns in the Dataset.

Observed Pattern	Completion sequence	Final completion year	Total (*n*)
1	1111111	3	670
2	0011111	5	531
3	0000000		365
4	0111111	4	55
5	1011111	5	27
6	1000000		13
7	0000001	10	5
8	0001111	7	4
9	0000011	9	4
10	0000111	8	4
11	1001111	7	1

The multinomial logistic regression analyses showed that only enrolment in the general vs. the vocational track, among the demographic and school-related variables, predicted higher odds for normal progression (completion) than non-completion (Table 2). Study track also predicted higher odds for delayed completion than non-completion, as did literacy problems and female gender. Among the psychological variables (Table 3), poorer general health predicted higher odds of delayed completion than non-completion. When adding GPA to the models, most effects turned non-significant, with enrollment in the general track and better general health still predicting higher odds of delayed completion than non-completion.

**Table 2:** Multinomial Regression of School-Related Variables and GPA on Completion Status.

*Completion*				
Intercept		.492		.003
Gender (female)	1.13 (.85 – 1.51)	.394	1.070 (.77 - 1.49)	.695
Family income level	0.87 (.70 - 1.07)	.191		
Track (general)	1.79 (1.30 - 2.46)	<.001	1.16 (.78 - 1.72)	.47
Educational plans	1.11 (.98 - 1.28)	.112		
Task solving abilities	1.06 (.92 - 1.22)	.414		
Literacy problems	0.60 (.35 - 1.01)	.053	0.87 (.48 – 1.60)	.658
Conflict w/classroom rules	1.02 (.92 - 1.12)	.804		
Being bullied	0.97 (.83 - 1.13)	.677		
GPA			1.82 (1.38 - 2.40)	<.001
*Delayed completion*				
Intercept		.687		<.001
Gender (female)	1.36 (1.01 - 1.83)	.044	1.05 (.75 - 1.47)	.784
Family income level	0.90 (.72 - 1.12)	.337		
Track (general)	2.89 (2.08 - 4.0)	<.001	1.71 (1.15 - 2.55)	.008
Educational plans	1.04 (.90 - 1.19)	.59		
Task solving abilities	1.10 (.95 - 1.30)	.184		
Literacy problems	0.37 (.21 - .66)	<.001	0.78 (.41 – 1.49)	.456
Conflict w/classroom rules	1.02 (.92 - 1.13)	.756		
Being bullied	0.86 (.72 - 1.03)	.108		
GPA			2.26 (1.70 - 3.0)	<.001

Reference category = non-completers. CI = confidence interval. Nagelkerke = 0.088 for first step, 0.092 for second step.

**Table 3:** Multinomial Regression of Psychological Variables and GPA on Completion Status.

*Normal progression*				
Intercept		.594		.004
General health	0.81 (.63 - 1.04)	.092	0.85 (.67 - 1.08)	.188
HSCL	1.01 (.74 - 1.39)	.994		
SWLS	1.05 (.90 - 1.22)	.564		
SOC	1.02 (.87 - 1.28)	.874		
RSA	0.95 (.72 - 1.25)	.696		
Social support	1.18 (.86 - 1.62)	.293		
Prevention focus	1.10 (.83 - 1.47)	.502		
Promotion focus	1.06 (.80 - 1.40)	.692		
PAQ masculine	1.32 (.95 - 1.83)	.094		
PAQ feminine	0.94 (.73 - 1.22)	.650		
Stressful Life Events	0.91 (.77 - 1.07)	.251		
Sleep time	0.98 (.87 - 1.11)	.758		
GPA			1.95 (1.57 – 2.41)	<.001
*Delayed*				
Intercept		.544		<.001
General health	0.71 (.55 - .92)	.011	0.74 (.57 - .95)	.017
HSCL	1.05 (.76 - 1.45)	.786		
SWLS	0.98 (.84 - 1.15)	.808		
SOC	0.93 (.73 - 1.17)	.51		
RSA	1.04 (.78 - 1.38)	.786		
Social support	1.24 (.89 - 1.72)	.204		
Prevention focus	0.93 (.70 - 1.25)	.640		
Promotion focus	1.32 (1.00 - 1.76)	.052		
PAQ masculine	1.05 (.76 - 1.47)	.754		
PAQ feminine	1.01 (.78 - 1.32)	.933		
Stressful Life Events	0.85 (.72 - 1.00)	.055		
Sleep time	1.05 (.93 - 1.20)	.433		
GPA			2.70 (2.16 – 3.37)	<.001

Reference category = non-completers. Nagelkerke = 0.036 for first step and 0.092 for second step. HSCL = Hopkins Symptom Checklist, SWLS = Satisfaction With Life Scale, SOC = Sense of Coherence, RSA = Resilience Scale Adults, PAQ = Personal Attributes Questionnaire.

In both models, higher GPA predicted higher odds of completion or delayed completion than non-completion. This effect was strongest for delayed completion. An ANOVA showed significant mean differences in GPA between the three groups, completion (M = 4.08, SD = .78), delayed completion (M = 4.30, SD = .73) and non-completion (M = 3.67, SD = .79), *F*(2, 1351) = 57.38, *p* < .001.

Given that GPA remained a single significant predictor in the completion model, and was a major contributor in the delayed completion model, we conducted additional exploratory regression analyses with GPA as the dependent variable. Correlational analyses showed that the associations between GPA and more stable (trait-like) variables were stronger than those between GPA and more situational (state-like) variables. In two regression models comparing their respective R-squared values, trait-like (i.e., family income level, problem solving abilities, learning problems, sense of coherence, resilience, personal attributes and motivational focus) accounted for 32% of the variance, whereas the state-like variables (conflict with classroom rules, bullying, general health, mental health, satisfaction with life, social support, stressful life events, and total sleep time) accounted for 15% of the variance. The incremental contribution of the state-like variables after accounting for the trait-like variables was minor (5%) but significant (*p* < .001).

## Discussion

The present study examined the predictive value of a broad set of demographic, school-related, psychological, and mental health variables for school completion. A discrete-time mixture analysis yielded three latent subgroups of students: normal progression (completion), delayed completion and non-completion. Females (vs. men), general educational track (vs. vocational), less literacy problems (vs. more), and better self-reported general health (vs. worse health) significantly predicted higher odds for completion or delayed completion 10 years after enrolment in upper secondary school than non-completion. However, school performance, as indicated through the Grade Point Average (GPA), was superior as its inclusion rendered most of these non-significant except enrollment in the general track and better general health that remained significant for predicting delayed completion. These results mirror the findings from a prior paper investigating five-year completion for the same sample [16], as well as the general extant literature on this topic. This paper also characterizes distinct empirical completion trajectories and identifies new predictors using a latent cluster-analytic approach. We caution against designating these classes as representatives of qualitatively different subpopulations in the educational setting, as differences between normal and delayed trajectories may reflect a statistical discretization of the variation in completion timing along a continuous scale. Nevertheless, we consider the latent approach a parsimonious probabilistic characterization of heterogeneous longitudinal completion patterns that better account for some patterns fitting less well with any of the trajectories. Another advantage of the latent mixture approach is its ability to reveal differences connected not only the timing or level of completion, but also to the predictor profile associated with the trajectories. In our data, this is reflected in GPA being a stronger predictor of delayed completion than of normal progression, a finding that was unexpected and is hard to capture through ordinary regression approaches.

Despite the findings for the normal progression students mimic previous findings, they are also informative by showing that the likelihood for completing one’s education is low after the first five years; hence, the stability in completion status is high. This study also reiterates the confidence in GPA as a major forecasting variable, as our inclusion of a broad set of school related, cognitive and psychological variables did not contribute beyond GPA. Our updated analyses thus confirm the contention that further improvement in the prediction of completion or dropout from school beyond the first 5-year period is unlikely. It thus consolidates the GPA's robustness as the single most important predictor of completion, and its importance from a prevention perspective. A practical prediction example makes it clear, as students who earn a GPA score of 2, 3, 4, or 5 increase their probability for completion from 47%, 65%, 79% to 89%, respectively. The school-, cognitive or psychological variables may still play an important role, if, for whatever reasons, it would be more feasible to raise the likelihood for completion through other routes than academic performance. The variables that correlated significantly with completion, i.e., literacy problems, task-solving abilities and health self-perception, are all factors that, despite relative stability, may be modifiable to targeted intervention and could achieve comparable prevention goals. Since these factors may be more viable for improvement through easier intervention than to raise students GPA, these factors are relevant to address as alternative routes to better grades.

Literacy problems may be related to school completion through both direct and indirect pathways, yet the non-significant association after including GPA in our analyses does not suggest a substantial direct effect. The indirect path through academic achievement may be more likely, because reading and writing difficulties may affect access to educational material as well as the ability to demonstrate knowledge through tests. Previous findings suggest that literacy problems do predict GPA [15], and a Finnish study showed that word-learning in 9^th^ grade predicted dropout from upper secondary education through school achievement in 9^th^ and 11^th^ grades [39]. However, our data did not allow us to perform mediation analyses investigating these mechanisms further.

A new and less expected finding was that GPA was more strongly associated with delayed completion than with the normal progression group. We have no obvious explanation for this, but one possibility may lie in barriers students may face, such as external challenges or situational factors. These barriers delay their progress, but the grades may represent an academic foundation that heightens their outlook for completion. Likewise, better general health and enrollment in the general track were also significant predictors of delayed completion compared to non-completion, which was not seen for the normal progression group. Better general health may imply fewer other issues in life preventing them from overcoming barriers and eventually completing studies despite initial struggles to stay on track. These findings highlight the importance of addressing both academic and non-academic factors, such as health and structural support, to reduce further delays and dropout for at-risk students.

Another somewhat novel finding is that factors representing trait-like conditions were substantially more strongly related to GPA than variables of a more state-like contextual nature. The variables that we treated as more trait-like indicators measured aspects related to motivational focus, resilience protective factors, literacy problems, and basic problem-solving skills. We classified literacy problems as trait-like because the scale captures relative enduring difficulties with decoding, reading comprehension, spelling, and written expression, overlapping the diagnostic criteria for Specific Learning Disorder in DSM 5 [40]. This is consistent with how specific learning disorders are typically characterized in the literature as relatively stable, compared to more state-like factors [41]. Yet, a relative stability does not mean immutability. There is indeed evidence for interventions to reduce such difficulties (e.g.[42]),. Since these factors seem much more important for GPA than state-like variables as satisfaction with life and stressful life events, they represent more effective indirect intervention routes to better grades, and hence, higher likelihood for completion than more state-like variables. Yet, we acknowledge that, as mental health fluctuates during adolescence [16] and may represent an obstacle, such factors should be screened for in combination of short- and long-term interventions.

### Strengths and limitations

One strength of the study is the high baseline response rate (~69%), which strengthens the generalizability. The gender and study-track distribution closely matched the population at the time of data collection, suggesting a representative sample on these statistics. The use of registry data for exam grades and school completion reduces risks of self-report biases, such as socially desirable responding. An issue was, however, that 76.5% had completed their education in 2015 in our study, which, compared to the Norwegian official statistics for students in Troms county in 2010, was ~ 67% [36]. The higher-than-expected completion rate in our sample may imply a more positive relationship with or attitude toward school than among those not participating. This is difficult to adjust for by weighting in the present study, as the completion rate among non-consenters is unknown and because the general assumption of 67% does not necessarily translate to our study. With that said, the potential bias is expected to be minor, as association coefficients are generally less biased than prevalence estimates in population-based studies [43]. Another possibility is the timing of pupils dropping out of school, as some quit early in the semester or do not start at all. These are consequently categorized as dropouts in the official statistics but would not be reachable during our data collection.

A prominent limitation was the low response rate in the follow-up questionnaires, thus prohibiting analyses of the extent to which changes over time in the measured variables might be more powerful. Attrition analyses showed that students with higher grades, female gender, and enrollment in the general track, tended to remain for the two follow-up waves. Non-responders did not differ from responders on most of the psychological, state-dependent variables measured at baseline. Most mental disorders have their onset in adolescence, which is a period characterized by fluctuating changes and less control over life [44]. For example, Dupéré and colleagues [45] found that 25% of the students dropping out reported heightened depressive symptoms three months before, and conversely, those remitting again reduced the risk of dropout. In our study, mental health was measured early in the first semester, which might mask the potential role of depression as beginning at a new school, meeting new people, might represent a new hope. Therefore, we could not examine the role of longitudinal changes in mental health. Future studies may thus benefit from being more successful in securing process-oriented or repeated measurements capable of detecting the fluctuating development and identity issues that characterize the adolescent period.

Class membership was analysed using the most likely class assignment rather than in a single-step model, which does not account for classification uncertainty. Although entropy was high, small biases in the estimated associations cannot be ruled out.

### Implications for practice and policy

The observation that previous academic performance predicts future school completion is neither novel nor groundbreaking. This relationship has been well-documented in prior research, and we believe it is also widely recognized by educators. A new finding is perhaps, though, the stability in the problem of completing school from five years onwards. A critical question may be how this knowledge can be transformed into a useful prevention intervention.

Norwegian students are participating in mandatory national testing in 5^th^, 8^th^, and 9^th^ grade [46]. The purpose of the national tests is to provide schools with insights into students’ competencies across three central subjects: reading, math, and English. The information obtained from these assessments serves as a foundation for formative evaluation and quality development at all levels of the educational system. These assessments may therefore be actively used by teachers to identify students who struggle and to consider targeted support. The practice of national testing has on the other hand been a topic of considerable debate, not only in Norway but across the Nordic countries. Critics argue that these tests influence teaching practices too much, leading educators to “teach to the test” rather than fostering learning. Additionally, concerns have been raised about the validity of these assessments amid challenges such as artificial intelligence and cheating. In response to these issues, the Norwegian government is currently working to reform national tests, aiming to make them more supportive of learning rather than solely tools for measuring and comparing results.

While we refrain from taking a definitive stance on the broader question of whether students’ skills should be assessed at a national level, we note that the overarching goal of such tests often appears to be improving average scores, particularly in international comparisons. Based on the present findings, there are more reasons than not to fully support efforts to enhance student learning and competence through these assessments. However, we emphasize that the primary focus should not be on grade inflation or superficial improvements in test scores. Instead, we advocate seeking strategies that address the underlying issue, i.e., how a low grade is a strong path to school dropout. Reducing this association should be a central priority in educational policy and practice.

Positive teacher-student relationships are important in this regard. Teachers and advisors in secondary education might benefit from alternative tools and strategies to help students with lower grades stay on track. Improved mandatory career guidance and information on different educational opportunities, along with enhanced psychological support, could help address this challenge. This includes further strengthening and promoting alternative pathways to obtaining a diploma. By ensuring such pathways are accessible and valued, students who may not thrive in traditional academic settings could still achieve meaningful qualifications and transition successfully into further education or the workforce. Teachers relate the increasing amount of theory to the dropout [47], making it harder for students with lower academic abilities to complete, and students who have quit school report discouragement and lack of support in their educational environment.

Qualitative research has shown that students who were discouraged and pessimistic had found school to be equally lacking in care and support as they had often experienced at home [48]. In contrast, optimistic and proactive students emphasized that supportive interactions with important others had made them believe in themselves and in alternative schooling. Indeed, good collaborative alliances with teachers reduce the risk of dropout [49]. Hence, above all, we believe that it is crucial to ensure that teachers and advisors have enough time to monitor, talk to, and find adequate solutions for students at risk – through dialogue and in collaboration with the adolescents themselves.

## Supporting information

S1 Table Descriptive statistics for included variables. (DOCX)

S2 Table Correlations between included variables. (DOCX)

## References

[1] Hjorth CF, Bilgrav L, Frandsen LS, Overgaard C, Torp-Pedersen C, Nielsen B, et al. Mental health and school dropout across educational levels and genders: A 4.8-year follow-up study. BMC Public Health. 2016;16:976. doi: 10.1186/s12889-016-3622-8 27627885 PMC5024430

[2] De Ridder KAA, Pape K, Johnsen R, Holmen TL, Westin S, Bjørngaard JH. Adolescent health and high school dropout: A prospective cohort study of 9000 Norwegian adolescents (the Young-HUNT). PLoS One. 2013;8(9):e74954. doi: 10.1371/journal.pone.0074954 24086408 PMC3781164

[3] Belfield CR, Levin HM. The price we pay: Economic and social consequences of inadequate education. Washington D.C.: Brookings Institution Press; 2007.

[4] Landgren V, Lindblad I, Nylander L, Gillberg C, Fernell E. Inability to start or complete upper secondary school strongly predicts unemployment and psychosocial and psychiatric adversities - A register-based follow-up study from southwestern Sweden. Acta Paediatr. 2024;113(10):2231–9. doi: 10.1111/apa.17264 38703013

[5] Putnam RD. Our kids: the American dream in crisis. New York: Simons & Schuster; 2015.

[6] IHME-CHAIN Collaborators. Effects of education on adult mortality: A global systematic review and meta-analysis. Lancet Public Health. 2024;9(3):e155–65. doi: 10.1016/S2468-2667(23)00306-7 38278172 PMC10901745

[7] Wilkinson R, Pickett K. The spirit level. London: Penguin Books; 2010.

[8] Markussen E. Videregående opplæring for (nesten) alle. Fagernes: Cappelen Damm AS; 2009.

[9] OECD. Education at a Glance 2011: OECD Indicators. OECD Publishing; 2011. Available from: 10.1787/eag-2011-en

[10] Statistics Norway. ssb.no. Upper secondary education, 2010. 2011 [cited 2022 Jun 21]. vailable from: https://www.ssb.no/en/utdanning/statistikker/vgu/aar/2011-05-05

[11] Statistics Norway. SSB. Completion rates of pupils in upper secondary education. 2025 [cited 2025 Dec 24]. Available from: https://www.ssb.no/en/utdanning/videregaende-utdanning/statistikk/gjennomforing-i-videregaende-opplaering

[12] Johansson B. Dropping out of school – a systematic and integrative research review on risk factors an interventions. Õrebro: Õrebro University; 2019.

[13] Gubbels J, van der Put CE, Assink M. Risk factors for school absenteeism and dropout: A meta-analytic review. J Youth Adolesc. 2019;48(9):1637–67. doi: 10.1007/s10964-019-01072-5 PMC6732159 31312979

[14] Winding TN, Nohr EA, Labriola M, Biering K, Andersen JH. Personal predictors of educational attainment after compulsory school: Influence of measures of vulnerability, health, and school performance. Scand J Public Health. 2013;41(1):92–101. doi: 10.1177/1403494812467713 23221378

[15] Sæle RG, Sørlie T, Nergård-Nilssen T, Ottosen KO, Goll CB, Friborg O. Demographic and psychological predictors of grade point average (GPA) in North-Norway: A particular analysis of cognitive/school-related and literacy problems. Educ Psychol. 2015;:1–22. doi: 10.1080/01443410.2014.998630

[16] Goll CB, Sørlie T, Friborg O, Ottosen KO, Sæle RG. Poorer self-reported mental health and general health among first year upper secondary school students do not predict school dropout: A five-year prospective study. Front Psychol. 2024;15:1304314. doi: 10.3389/fpsyg.2024.1304314 38440235 PMC10911125

[17] Dohrenwend BP. The role of adversity and stress in psychopathology: Some evidence and its implications for theory and research. J Health Soc Behav. 2000;41(1):1–19. doi: 10.2307/2676357 10750319

[18] Kessler RC. The effects of stressful life events on depression. Annu Rev Psychol. 1997;48:191–214. doi: 10.1146/annurev.psych.48.1.191 9046559

[19] Luthar SS. Resilience in Development: A Synthesis of Research across Five Decades. Developmental Psychopathology. In: Cohen DCDJ. Developmental psychopathology, Vol 3: Risk, disorder, and adaptation (2nd ed.). Hoboken, NJ, US: John Wiley & Sons Inc; 2015. 739–95. doi: 10.1002/9780470939406.ch20

[20] Garmezy N. Children in poverty: Resilience despite risk. Psychiatry. 1993;56(1):127–36. doi: 10.1080/00332747.1993.11024627 8488208

[21] Antonovsky A. Unraveling the mystery of health: How people manage stress and stay well. San Francisco, CA, US: Jossey-Bass; 1987. 218.

[22] Antonovsky A. The structure and properties of the sense of coherence scale. Soc Sci Med. 1993;36(6):725–33. doi: 10.1016/0277-9536(93)90033-z 8480217

[23] Super S, Wagemakers MAE, Picavet HSJ, Verkooijen KT, Koelen MA. Strengthening sense of coherence: Opportunities for theory building in health promotion. Health Promot Int. 2016;31(4):869–78. doi: 10.1093/heapro/dav071 26208654

[24] Bakken A. Ungdata 2021. National results. Oslo: NOVA/OsloMet. 2021. https://oda.oslomet.no/oda-xmlui/handle/11250/2767874

[25] Bakken A. Ungdata 2022. National results. Oslo: NOVA/OsloMet. 2022. https://oda.oslomet.no/oda-xmlui/handle/11250/3011548

[26] Madsen OJ. Generasjon prestasjon. Hva er det som feiler oss?. Universitetsforlaget; 2018.

[27] Gustafsson JE, Allodi MW, Åkerman A, Eriksson C, Eriksson L, Fischbein S. School, learning and mental health: A systematic review. Stockholm: Health Committee, Royal Swedish Academy of Sciences; 2010.

[28] Sagatun A, Heyerdahl S, Wentzel-Larsen T, Lien L. Mental health problems in the 10th grade and non-completion of upper secondary school: The mediating role of grades in a population-based longitudinal study. BMC Public Health. 2014;14:16. doi: 10.1186/1471-2458-14-16 24406098 PMC3905670

[29] Strand BH, Dalgard OS, Tambs K, Rognerud M. Measuring the mental health status of the Norwegian population: A comparison of the instruments SCL-25, SCL-10, SCL-5 and MHI-5 (SF-36). Nord J Psychiatry. 2003;57(2):113–8. doi: 10.1080/08039480310000932 12745773

[30] Diener E, Emmons RA, Larsen RJ, Griffin S. The satisfaction with life scale. J Pers Assess. 1985;49(1):71–5. doi: 10.1207/s15327752jpa4901_13 16367493

[31] Friborg O, Hjemdal O, Martinussen M, Rosenvinge JH. Empirical support for resilience as more than the counterpart and absence of vulnerability and symptoms of mental disorder. J Individ Differ. 2009;30(3):138–51. doi: 10.1027/1614-0001.30.3.138

[32] Lockwood P, Jordan CH, Kunda Z. Motivation by positive or negative role models: Regulatory focus determines who will best inspire us. J Pers Soc Psychol. 2002;83(4):854–64. doi: 10.1037/0022-3514.83.4.854 12374440

[33] Higgins ET. Beyond pleasure and pain. Am Psychol. 1997;52(12):1280–300. doi: 10.1037//0003-066x.52.12.1280 9414606

[34] Spence JT, Helmreich RL. Masculinity & femininity. Austin: University of Texas Press; 1978.

[35] Roenneberg T, Wirz-Justice A, Merrow M. Life between clocks: Daily temporal patterns of human chronotypes. J Biol Rhythms. 2003;18(1):80–90. doi: 10.1177/0748730402239679 12568247

[36] Statistics Norway. ssb.no. Throughput of pupils in upper secondary education, 2010-2015. 2016 [cited 2022 Jun 22]. Available from: https://www.ssb.no/utdanning/statistikker/vgogjen/aar/2016-06-02?fane=tabell&sort=nummer&tabell=268111

[37] Muthén LK, Muthén BO. Mplus User’s Guide. 8 ed. Los Angeles, CA: Muthén & Muthén; 1998.

[38] IBM Corp. IBM SPSS Statistics for Windows. Armonk, NY: IBM Corp; 2019.

[39] Hakkarainen AM, Holopainen LK, Savolainen HK. A five-year follow-up on the role of educational support in preventing dropout from upper secondary education in Finland. J Learn Disabil. 2015;48(4):408–21. doi: 10.1177/0022219413507603 24150709

[40] American Psychiatric Association. Diagnostic and statistical manual of mental disorders: DSM-5. Washington, D.C.: American Psychiatric Association; 2013.

[41] Snowling MJ, Hulme C. Annual research review: The nature and classification of reading disorders – a commentary on proposals for DSM-5. J Child Psychol Psychiatry. 2012;53(5):593–607. doi: 10.1111/j.1469-7610.2011.02495.x PMC3492851 22141434

[42] Mitchell JL, Yablonski M, Stone HL, Jimenez M, Takada ME, Tang KA, et al. The balance between stability and plasticity of the visual word form area in dyslexia. Nat Commun. 2025;17(1):368. doi: 10.1038/s41467-025-67054-3 41372176 PMC12796487

[43] Stormark KM, Heiervang E, Heimann M, Lundervold A, Gillberg C. Predicting nonresponse bias from teacher ratings of mental health problems in primary school children. J Abnorm Child Psychol. 2008;36(3):411–9. doi: 10.1007/s10802-007-9187-3 18161021

[44] Patel V, Flisher AJ, Hetrick S, McGorry P. Mental health of young people: A global public-health challenge. Lancet. 2007;369(9569):1302–13. doi: 10.1016/s0140-6736(07)60368-7 17434406

[45] Dupéré V, Dion E, Nault-Brière F, Archambault I, Leventhal T, Lesage A. Revisiting the link between depression symptoms and high school dropout: Timing of exposure matters. J Adolesc Health. 2018;62(2):205–11. doi: 10.1016/j.jadohealth.2017.09.024 29195763

[46] The Norwegian Directorate for Education and Training. Rammeverk for nasjonale prøver [Framework for national tests]. The Norwegian Directorate for Education and Training; 2022. Available from: https://www.udir.no/eksamen-og-prover/prover/rammeverk-for-nasjonale-prover2/

[47] Ottosen KO, Goll CB, Sørlie T. The multifaceted challenges in teacher-student relationships: A qualitative study of teachers’ and principals’ experiences and views regarding the dropout rate in Norwegian upper-secondary education. Scand J Educ Res. 2016;:1–15. doi: 10.1080/00313831.2016.1147069

[48] Ottosen KO, Goll CB, Sørlie T. ‘From a sense of failure to a proactive life orientation’: First year high school dropout experiences and future life expectations in Norwegian youth. International Social Work. 2017;62(2):684–98. doi: 10.1177/0020872817746225

[49] Noble RN, Heath N, Krause A, Rogers M. Teacher-student relationships and high school drop-out: applying a working alliance framework. Can J Sch Psychol. 2021;36(3):221–34. doi: 10.1177/0829573520972558

