# Supplementary material for: Completing upper secondary school: Results from a ten-year longitudinal study in Norway
Source: PLoS One. 2026 Nov 6;21(11):e0361808. doi: 10.1371/journal.pone.0361808 (PMC13742913; doi:10.1371/journal.pone.0361808)
Supplement: Supplementary file 1 [file pone.0361808.s001.docx]

**S1 Table.**

Descriptive statistics for included variables

| Variable | *M* / % | *SD* | *N* |
| --- | --- | --- | --- |
| Class 1 = completed  Class 2 = non-completed  Class 3 = delayed | 40.0  23.3  36.7 |  | 1677  1677  1677 |
| Gender (female) | 48 |  | 1676 |
| Our family's economy (1-4) | 2.61 | 0.68 | 1646 |
| Track (general) | 45.6 |  | 1666 |
| Plans for education (1 = not decided, 2 = upper secondary, 3 = Bachelor level, 4 = Master level) | 2.46 | 1.17 | 1570 |
| Task solving abilities (1-5) | 4.49 | 1.15 | 1619 |
| Literacy problems (0-1) | 0.24 | 0.29 | 1624 |
| Conflict with rules in the classroom (1-5) | 2.40 | 1.46 | 1639 |
| Bullied or harassed at school (1-5) | 1.27 | 0.84 | 1618 |
| General health (1-5) | 1.80 | 0.69 | 1631 |
| Hopkins symptom (1-5) | 1.58 | 0.64 | 1610 |
| Satisfaction of life (1-5) | 3.20 | 1.37 | 1510 |
| Sense of coherence (1-5) | 4.24 | 0.97 | 1580 |
| RSA mean total (1-5) | 3.94 | 0.73 | 1536 |
| Social support mean (1-5) | 4.42 | 0.58 | 1636 |
| Prevention focus (1-5) | 3.05 | 0.71 | 1528 |
| Promotion focus (1-5) | 3.68 | 0.72 | 1528 |
| PAQ Masculine (1-5) | 3.36 | 0.63 | 1530 |
| PAQ Feminine (1-5) | 3.79 | 0.67 | 1530 |
| Stressful life events (0-6) | 0.81 | 0.95 | 1567 |
| Total Sleep Time Workdays | 6.81 | 1.24 | 1509 |
| GPA (0-6) | 4.09 | 0.80 | 1362 |

*Note*. Percentages are given for categorical variables (classes, gender, track).
