# Supplementary material for: Completing upper secondary school: Results from a ten-year longitudinal study in Norway
Source: PLoS One. 2026 Nov 6;21(11):e0361808. doi: 10.1371/journal.pone.0361808 (PMC13742913; doi:10.1371/journal.pone.0361808)
Supplement: Supplementary file 2 [file pone.0361808.s002.docx]

**S2 Table.**

| Variable | 1 | 2 | 3 | 4 | 5 | 6 | 7 | 8 | 9 | 10 | 11 | 12 | 13 | 14 | 15 | 16 | 17 | 18 | 19 | 20 | 21 | 22 |
| --- | --- | --- | --- | --- | --- | --- | --- | --- | --- | --- | --- | --- | --- | --- | --- | --- | --- | --- | --- | --- | --- | --- |
| 1. Completion status^1^ |  |  |  |  |  |  |  |  |  |  |  |  |  |  |  |  |  |  |  |  |  |  |
| 2. Class^2^ | -.030 |  |  |  |  |  |  |  |  |  |  |  |  |  |  |  |  |  |  |  |  |  |
| 3. Gender^3^ | -.092 | -.038 |  |  |  |  |  |  |  |  |  |  |  |  |  |  |  |  |  |  |  |  |
| 4. Family income level | .009 | .034 | -.021 |  |  |  |  |  |  |  |  |  |  |  |  |  |  |  |  |  |  |  |
| 5. Track^4^ | -.178 | -.111 | .135 | -.142 |  |  |  |  |  |  |  |  |  |  |  |  |  |  |  |  |  |  |
| 6. Educational plans | .102 | .021 | -.105 | .173 | -.334 |  |  |  |  |  |  |  |  |  |  |  |  |  |  |  |  |  |
| 7. Task solving abilities | .091 | .043 | -.066 | .163 | -.220 | .300 |  |  |  |  |  |  |  |  |  |  |  |  |  |  |  |  |
| 8. Literacy problems | -.130 | -.079 | .150 | -.151 | .294 | -.201 | -.443 |  |  |  |  |  |  |  |  |  |  |  |  |  |  |  |
| 9. Conflict with rules | -.051 | -.018 | .146 | -.024 | .162 | -.100 | -.233 | .149 |  |  |  |  |  |  |  |  |  |  |  |  |  |  |
| 10. Being bullied | -.061 | -.041 | .097 | -.047 | .104 | -.059 | -.143 | .177 | .097 |  |  |  |  |  |  |  |  |  |  |  |  |  |
| 11. General health | -.104 | -.056 | -.107 | -.192 | .236 | -.169 | -.238 | .202 | .107 | .073 |  |  |  |  |  |  |  |  |  |  |  |  |
| 12. HSCL | -.040 | -.020 | -.185 | -.159 | .042 | -.057 | -.215 | .216 | .079 | .241 | .335 |  |  |  |  |  |  |  |  |  |  |  |
| 13. SWLS | -.052 | -.042 | -.114 | -.236 | .094 | -.133 | -.218 | .107 | .110 | .146 | .368 | .478 |  |  |  |  |  |  |  |  |  |  |
| 14. SOC | .047 | .022 | .166 | .178 | -.124 | .090 | .280 | -.164 | -.194 | -.176 | -.342 | -.574 | -.565 |  |  |  |  |  |  |  |  |  |
| 15. RSA | .054 | .058 | -.002 | .209 | -.155 | .156 | .238 | -.200 | -.129 | -.164 | -.301 | -.355 | -.507 | .449 |  |  |  |  |  |  |  |  |
| 16. Social support | .085 | .049 | -.109 | .192 | -.163 | .175 | .270 | -.242 | -.109 | -.211 | -.235 | -.368 | -.396 | .359 | .421 |  |  |  |  |  |  |  |
| 17. Prevention | -.001 | -.009 | -.183 | -.051 | -.002 | -.014 | -.122 | .107 | -.003 | .088 | .145 | .399 | .261 | -.407 | -.196 | -.154 |  |  |  |  |  |  |
| 18. Promotion | .060 | .065 | -.146 | .141 | -.261 | .231 | .202 | -.174 | -.147 | -.099 | -.152 | .034 | -.109 | .061 | .277 | .151 | .421 |  |  |  |  |  |
| 19. PAQ Masculine | .082 | .028 | .091 | .250 | -.232 | .283 | .328 | -.261 | -.053 | -.088 | -.356 | -.323 | -.436 | .411 | .467 | .372 | -.226 | .282 |  |  |  |  |
| 20. PAQ Feminine | .053 | .035 | -.279 | .112 | -.161 | .144 | .203 | -.183 | -.185 | -.078 | -.087 | .016 | -.148 | .096 | .258 | .274 | .112 | .325 | .354 |  |  |  |
| 21. SLE | .054 | -.041 | -.118 | -.052 | .086 | .007 | -.040 | .061 | .077 | .039 | .148 | .182 | .117 | -.217 | -.021 | -.036 | .123 | .045 | .043 | .066 |  |  |
| 22. Sleep Time | .055 | .058 | -.044 | .095 | -.179 | .148 | .129 | -.058 | -.187 | -.085 | -.168 | -.097 | -.159 | .205 | .094 | .093 | -.048 | .135 | .106 | .040 | -.104 |  |
| 23. GPA | .200 | .123 | -.293 | .149 | -.587 | .399 | .434 | -.438 | -.233 | -.140 | -.198 | -.009 | -.063 | .075 | .176 | .220 | .005 | .310 | .259 | .249 | .021 | .182 |

Correlations between included variables

*Note*. Spearman’s rho is calculated for correlations between dichotomous variables, otherwise Pearson’s r. Correlations above .048 correspond to *p* < .05, above .063 correspond to *p* < .01, and above 0.08 correspond to *p* < .001. ^1^ 0 = non-completed, 1 = completed ^2^ 1 = completed, 2 = non-completed, 3 = delayed, ^3^ 0 = female and 1 = male, ^4^ 0 = general and 1 = vocational track. HSCL = Hopkins Symptom Checklist, SWLS = Satisfaction With Life Scale, SOC = Sense of Coherence, RSA = Resiliense Scale Adults, PAQ = Personal Attributes Questionnaire, SLE = Stressful Life Events, GPA = Grade Point Average.
