# Supplementary material for: Completing upper secondary school: Results from a ten-year longitudinal study in Norway
Source: PLoS One. 2026 Nov 6;21(11):e0361808. doi: 10.1371/journal.pone.0361808 (PMC13742913; doi:10.1371/journal.pone.0361808)
Supplement: Supplementary file 4 [file pone.0361808.s005.docx]

*We thank the editor and the reviewers for their valuable comments. Since the editor’s summary clearly captures and specifies the reviewers’ comments, we organized our main response according to the editor’s bullet list. Additional comments not covered there are addressed separately below.*

**Editor Comments:**
MAJOR ISSUES
1. Justification of the latent trajectory approach
The central contribution of the manuscript rests on the identification of three latent trajectories: normal progression, delayed completion, and non-completion. However, the manuscript does not sufficiently demonstrate that these classes represent substantively distinct educational trajectories rather than a statistical discretization of a continuous distribution of completion times.

The distinction between “normal progression” and “delayed completion” appears to be primarily temporal. As currently presented, it remains unclear whether the latent class solution is uncovering genuinely distinct populations of students or merely partitioning variation in the timing of graduation.

The revised manuscript should therefore:
- Provide a stronger conceptual rationale for the use of latent classes.
- Clarify how the normal and delayed completion groups differ in terms of actual completion timing.
- Present descriptive information on the distribution of completion times within each latent class.
- Discuss why the latent trajectory approach is preferable to a more direct classification based on observed completion timing.
- Explain what substantive insight is gained from the latent class solution that could not be obtained from observed time-to-completion data.

*We agree that the manuscript does not justify this sufficiently and have revised the Methods/Statistical analyses section (p.11) accordingly. First, we do not consider the latent variable approach as sufficient to indicate distinct subpopulations with respect to completion, which require more rigorous testing and validation of theoretical, practical, or clinical meaningful differences that identified trajectories may incur. We rather wanted to examine if empirically distinct temporal patterns exist, and if they can meaningfully account for the heterogeneity in educational progression. Heterogeneity was a characteristic of the recorded completion data, as completion status was a reversible variable that could change its value several times through the observation period. For example, students could complete their curriculum at 3 years, but then switch to another curriculum that they did or did not complete. Because of this, we replaced “discrete-time survival mixture model” with “longitudinal latent class mixture model” to avoid misinterpretation of the completion-status coding. An observed time approach would be problematic because it forces a potentially misleading classification. The LCA, by contrast, determines latent class membership based on model-based posterior probabilities of belonging to each class, so the decision becomes probability-based rather than deterministic. Because the trajectories were based on completion patterns that showed some variation in completion time, we believe the LCA approach can better summarize heterogeneous completion patterns through more parsimonious common latent trajectories that describe students’ progress in the educational system. If the covariates we regressed on the latent classes show very different results, that would strongly indicate the usefulness of this method, which ordinary regression would miss.*

*We have included descriptive information of the 11 observed completion patterns, together with the subsample size and year of final completion, in a new Table 1 (p.14). We also reconsider the added value of using a latent class approach in the Discussion (p.18). We hope these amendments sufficiently describe the methodological choices.*

2. Use of longitudinal information and survival modelling
The manuscript is presented as a ten-year longitudinal study and employs a discrete-time survival mixture model. However, most substantive conclusions are ultimately derived from multinomial logistic regressions predicting latent class membership.
As a result, the temporal dimension of the data appears underutilized. The manuscript would benefit from a more explicit justification of this analytical strategy.

Specifically, the authors should:
- Clarify why multinomial logistic regression was selected as the primary inferential framework following trajectory identification.
- Explain how the chosen approach compares conceptually and analytically with alternative event-history approaches.
- Discuss why the observed completion process was not analysed more directly using event-history or competing-risk frameworks based on educational outcomes over time.
- Consider adding descriptive survival information (e.g., annual completion and non-completion patterns, cumulative completion probabilities, or related summaries) to help readers understand the temporal dynamics underlying the trajectory solution.

I am not requiring a complete reanalysis of the data. However, a substantially stronger justification of the methodological choices is necessary.

*Prediction of educational completion was the main objective of this paper; therefore, we used logistic (or multinomial) regression with dichotomous completion status as the outcome. In this respect, the introductory latent trajectory analysis was a novel approach for examining how the various observed completion patterns can be accounted for by some fewer general trajectories. Since the latent trajectories are representations of different longitudinal completion patterns, the latent class membership variable was the outcome of interest. Please find an extended description of the rationale for this analysis approach in the Methods/Analyses section (p.11), which we hope is sufficient.*

*Regarding the potential alternative use of event-history approaches, we refer to the amendments to the Method/Analyses section. The major issue is the reversibility of completion status, which creates multiple completion patterns that a latent class approach can handle better by identifying latent trajectories that capture (potentially) qualitatively different temporal patterns over 10 years. Regression of covariates on these trajectories thus indirectly indicates longitudinal covariation. We have included the annual completion in Table 1.*

3. Educational pathways and interpretation of completion trajectories
The manuscript includes students from both general and vocational tracks. It is not sufficiently clear whether differences in timing of completion may partly reflect structural differences between educational pathways rather than distinct latent student trajectories.

The authors should therefore:
- Provide a clearer description of the Norwegian upper secondary education system for an international readership.
- Explain the duration, objectives, and qualifications associated with the general and vocational tracks.
- Discuss whether differences in institutional organisation, programme structure, or expected completion patterns across tracks may influence the latent trajectory solution.
- Clarify the extent to which the delayed completion group may reflect educational pathway characteristics rather than individual-level heterogeneity.

*This is a sharp second thought, as several vocational pathways indeed progress over four rather than three years, and we have therefore extended the description of upper secondary education in Norway (beginning of the Methods section). It is correct that such factors will influence the form of the latent trajectories. One solution is to add track as a covariate to adjust the trajectories; however, we find it more informative to understand how the trajectories unfold as is. This is also standard practice, which is later extended by introducing covariates to understand their association with the trajectories* (Nylund-Gibson & Choi, 2018)*. This approach also offers a more intuitive and direct understanding of how, for example, educational track is related to different trajectories.*

4. Selection bias and external validity
The manuscript acknowledges that completion rates in the study sample were higher than those observed for the corresponding county population.

This issue deserves a more thorough treatment.
Please:
- Expand the discussion of possible selection effects.
- Clarify the extent to which the study sample can be considered representative of the wider cohort.
- Discuss implications for external validity and generalisability.
- Explain whether weighting procedures were considered and, if not, why they were not feasible.

*The higher completion rate in our data than in the county’s registered data may reflect selection bias, such as students (or parents) who consented differing from non-consenters in ways associated with school completion, or that students who dropped out of school before the data collection were non-contactable. These possibilities may have biased the observed completion rate relative to the official statistics.*

*The first aspect was already discussed in Strengths and Limitations, but we have elaborated on it and mentioned it in the Methods section. Weighting was unfortunately judged unfeasible, and since we did not have registry completion data for all schools in Troms county during the observation period, we did not consider weighting sufficiently justified. A suitable weighting variable taking both respondents and non-respondents into account, ideally associated with both participation and the outcome variable, was unavailable to us. Nor was registry data for performing meaningful population-weighted adjustments related to, e.g., family background, school support), beyond the comparisons already reported (gender, track).*

5. Attrition and follow-up participation
Although the principal outcome was obtained through registry data, the study experienced substantial attrition across questionnaire waves.

The revised manuscript should include:
- An attrition analysis comparing participants who remained in later waves with those who did not.
- A discussion of whether attrition may have affected the interpretation of the broader longitudinal project.
- Clarification of why follow-up questionnaire data were not incorporated into the present analyses.

*We did not include the second and third waves in the analysis because of the very low response rate. The mentioning of the follow-up studies are primarily included for transparency. However, we have added attrition analyses comparing participants who stayed in the study at T2 and/or T3 with those who only responded to the baseline survey. This analysis is reported in the Methods section (p.12), and mentioned also in the discussion (p.22).*

*While we wholeheartedly agree that including the follow-up studies would give us considerably more insight into possible explanations for the dropout patterns in our sample, we think the value of the longitudinal registry data still justifies the study.*

ADDITIONAL ISSUES

6. Interpretation of GPA and literacy problems
The manuscript shows that GPA dominates the multivariable models and that literacy problems lose statistical significance after GPA is introduced.

Please discuss more explicitly:
- Whether GPA may mediate the relationship between literacy problems and educational completion.
- Whether literacy difficulties contribute indirectly to completion outcomes through academic achievement.
- The implications of this interpretation for educational interventions.

*We agree that it would be an interesting matter. Unfortunately, we only have baseline data for the predictors (as mentioned above). Mediation studies should ideally be measured after the baseline. The expected added value of including mediation analyses, weighed against the increased length of the paper, is likely minimal. We therefore prefer to pursue this issue in another study.*

7. Classification of “trait-like” and “state-like” factors
The rationale for classifying literacy problems as a trait-like factor requires clarification.

Please provide a stronger conceptual justification for this classification and discuss whether literacy-related difficulties may be amenable to educational intervention and change over time.

*We classified literacy problems as trait-like because the scale captures relative enduring difficulties with decoding, reading comprehension, spelling, and written expression, overlapping the diagnostic criteria for Specific Learning Disorder in DSM 5* (American Psychiatric Association, 2013)*. This is consistent with how specific learning disorders are typically characterized in the literature as relatively stable, compared to more state-like factors* (Snowling & Hulme, 2021)*. The relatively high stability does not mean immutability, though, as interventions aimed at reducing such difficulties may work* (Mitchell et al., 2025)*, but we nevertheless consider the current framing as defensible. An amendment to the discussion has nevertheless been made.*

8. Measures
Please provide the number of items included in the literacy problems scale and ensure that all scales are described with sufficient detail to allow replication.

*Thank you for pointing this out. We have updated the Measure section.*

9. Accessibility for an international audience
The paper would benefit from additional contextual information regarding the Norwegian educational system, particularly for readers unfamiliar with the distinction between upper secondary, vocational

*Thank you, this is now provided in the Methods section.*

10. Data availability and reproducibility
The current Data Availability Statement indicates that the minimal dataset will be deposited in DataverseNO and that a DOI will be provided. However, it remains unclear whether the dataset and associated documentation will be sufficiently available to allow independent verification and replication of the analyses.

Please:

- Update the Data Availability Statement in accordance with PLOS ONE requirements.
- Provide the repository DOI if already available, or clearly explain its status and expected availability upon publication.
- Specify which variables and data files will be made available.
- Clarify whether the data underlying all analyses reported in the manuscript will be accessible without restriction, and if any legal or ethical constraints apply.
- Provide information on the availability of any analysis code, syntax files, or scripts used for data management and statistical analyses, as this would substantially enhance reproducibility.

*The data set is now openly available and an updated Data Availability Statement and DOI are provided.*

**Reviewer #1:**

**General comment:**

The article uses longitudinal data to examine upper secondary school completion in Norway over a ten-year period. This constitutes the article's major contribution, as longitudinal analysis provides a better understanding of the sequence of events in individuals' lives and the associated factors—factors that may be either fixed characteristics or ones that vary over the observation period. This longitudinal approach is well-suited to the analysis of educational pathways, given that educational events unfold over the course of successive years or grade levels.

**Specific comments:**

They focus primarily on the study's methodology. Several methodological limitations are not sufficiently discussed, and certain methodological choices raise questions and require further clarification from the authors.

**1) Selection bias:**

In addition to the baseline survey conducted in 2010 (wave 1), the study carried out two further waves in 2013 and 2020. Unfortunately, the very low response rates for waves 2 and 3 make it impossible to reconstruct true panel data or to fully leverage the richness of such data (e.g., measurements of time-varying variables). The response rate for the first wave—the data finally used I the paper—is also low (69%). Beyond the low response rate, the data reveal a selection bias; the authors acknowledge (pp. 17–18) that the completion rate for their sample is significantly higher than the average for the Troms County (76.5% vs. 67%, respectively). The study population thus appears more inclined towards schooling and likely comes from a family background that is more supportive of schooling. Consequently, this selection bias should have been discussed in greater depth: (i) one way to address this would be to adjust the sample using weights based on specific characteristics of the entire population of the County to ensure the representativity of the findings; (ii) otherwise, the limitation must be discussed in the methodology section, clarifying that the study involves a specific population within Troms County and that the results cannot be generalized to the county as a whole.

*We agree that the low response rates on waves 2 and 3 are unfortunate and undermine full advantage of the longitudinal design. Our analysis instead leverages the longitudinal structure of the outcome, while predictor data were limited to the baseline. However, we have revised the manuscript with this in mind (see comments above).*

*Regarding response rate (to the first wave), we respectfully disagree that 69% should be characterized as a low response rate. In a large European comparison with children and adolescents, responses ranged from 18.9 to 91.2 %* (Berra et al., 2007)*. School-based surveys ranged from 69 to 91.2 %. Another, newer study on survey research, average response rates were reported to have increased from 53 % in 2010, when our survey was conducted, to 68 % in 2020* (Holtom et al., 2022)*. Our response rate of 69% is therefore within the typical range of school-based surveys, and high compared to general surveys.*

**2) Statistical analyses:**

The key advantage of using longitudinal data is leveraging time, a key variable to consider when modeling the likelihood of the outcome variable. The authors fitted a discrete-time survival mixture model to identify three (3) latent trajectories of completion status. These latent variables are then used as dependent variable in multinomial logistic models. Apart of this objective of highlighting latent variables and identifying predictors of the different school completion groups, it is worth illustrating “survival” or “failure” table of the 1,676 enrolled in the study in 2010. A good number of summary statistics from the use the discrete time analysis is insightful for the education authorities and for sound education policies: e.g., the classes or years after upper secondary school beginning where there are mor risks of dropouts and cumulative risks of dropouts according to different individual and family background information.

Beyond the low response rate, the data reveal a selection bias. The study population thus appears more inclined towards schooling and likely comes from a family background that is more supportive of schooling. One way to address this would be to adjust the sample using weights based on specific characteristics of the entire population of the County to ensure the representativity of the findings. Otherwise, the limitation must be discussed in the methodology section, clarifying that the study involves a specific population within Troms County and that the results cannot be generalized to the county as a whole.
It is worth illustrating “survival” or “failure” table of the 1,676 enrolled in the study in 2010. A good number of summary statistics from the use the discrete time analysis is insightful for the education authorities and for sound education policies: e.g., the classes or years after upper secondary school beginning where there are mor risks of dropouts and cumulative risks of dropouts according to different individual and family background information.

*See responses above.*

**Reviewer #2:**

I’ll start with a minor point: it would be helpful if the section on the questions used also stated how many items are in the ‘Literacy problems’ scale.

*Thank you for pointing this out. We have now included it in the Methods section.*

As a non-Norwegian, I find it somewhat difficult to grasp the difference between the general and vocational tracks. Another point that might be helpful for readers unfamiliar with the Norwegian school system is: what does the upper secondary school curriculum cover, and for which professions does it provide qualifications?

*We have included a short description of the Norwegian school system.*

The article compares the data from the three distinct main groups with one another. This is legitimate and is likely to be of interest to most readers, particularly as the study’s design does not allow for an analysis of why someone belongs to the ‘Completion’ group or the ‘Delayed completion’ group. Whilst the statistical analysis suggests that this was primarily due to the previously achieved GPA, it does not explain why the other criteria surveyed play virtually no role. However, the strong negative correlation between GPA and literacy problems does leave room for speculation here. Unfortunately, there is no attrition analysis, which might have provided some insight into this. After all, data is available for most study participants at the first measurement point. So one could (at least in a separate analysis) investigate whether those who completed the questionnaires at two or more measurement points differ from those who only completed the initial questionnaire.

*See response under the editor’s point 5 above.*

One final suggestion for the authors: in the discussion on page 17, you classify literacy problems as trait-like factors – why is that? If literacy problems were defined as state-like, the obvious question would be: are there elements of upper secondary education designed to bring about a change in this state? And then the question would arise as to the extent to which the levels of the trait-like factors in the three main groups have changed over time.

*See response under the editor’s point 7 above.*

**References**

American Psychiatric Association. (2013). *Diagnostic and statistical manual of mental disorders: DSM-5*. American Psychiatric Association.

Berra, S., Ravens-Sieberer, U., Erhart, M., Tebé, C., Bisegger, C., Duer, W., von Rueden, U., Herdman, M., Alonso, J., Rajmil, L., & the European KIDSCREEN group [kidscreen]. (2007). Methods and representativeness of a European survey in children and adolescents: The KIDSCREEN study. *BMC Public Health*, *7*(1), 182. https://doi.org/10.1186/1471-2458-7-182

Holtom, B., Baruch, Y., Aguinis, H., & A Ballinger, G. (2022). Survey response rates: Trends and a validity assessment framework. *Human Relations*, *75*(8), 1560–1584. https://doi.org/10.1177/00187267211070769

Mitchell, J. L., Yablonski, M., Stone, H. L., Jimenez, M., Takada, M. E., Tang, K. A., Tran, J. E., Chou, C., & Yeatman, J. D. (2025). The balance between stability and plasticity of the visual word form area in dyslexia. *Nature Communications*, *17*(1), 368. https://doi.org/10.1038/s41467-025-67054-3

Nylund-Gibson, K., & Choi, A. Y. (2018). Ten frequently asked questions about latent class analysis. *Translational Issues in Psychological Science*, *4*(4), 440–461. https://doi.org/10.1037/tps0000176

Snowling, M. J., & Hulme, C. (2021). Annual Research Review: Reading disorders revisited – the critical importance of oral language. *Journal of Child Psychology and Psychiatry*, *62*(5), 635–653. https://doi.org/10.1111/jcpp.13324
