# Supplementary material for: Completing upper secondary school: Results from a ten-year longitudinal study in Norway
Source: PLoS One. 2026 Nov 6;21(11):e0361808. doi: 10.1371/journal.pone.0361808 (PMC13742913; doi:10.1371/journal.pone.0361808)
Supplement: Supplementary file 3 [file pone.0361808.s003.docx]

**Review of**

**Completing upper secondary school: Results from a ten-year longitudinal study in Norway**

**General comment:**

The article uses longitudinal data to examine upper secondary school completion in Norway over a ten-year period. This constitutes the article's major contribution, as longitudinal analysis provides a better understanding of the sequence of events in individuals' lives and the associated factors—factors that may be either fixed characteristics or ones that vary over the observation period. This longitudinal approach is well-suited to the analysis of educational pathways, given that educational events unfold over the course of successive years or grade levels.

**Specific comments:**

They focus primarily on the study's methodology. Several methodological limitations are not sufficiently discussed, and certain methodological choices raise questions and require further clarification from the authors.

**1) Selection bias:**

In addition to the baseline survey conducted in 2010 (wave 1), the study carried out two further waves in 2013 and 2020. Unfortunately, the very low response rates for waves 2 and 3 make it impossible to reconstruct true panel data or to fully leverage the richness of such data (e.g., measurements of time-varying variables). The response rate for the first wave—the data finally used I the paper—is also low (69%). Beyond the low response rate, the data reveal a selection bias; the authors acknowledge (pp. 17–18) that the completion rate for their sample is significantly higher than the average for the Troms County (76.5% vs. 67%, respectively). The study population thus appears more inclined towards schooling and likely comes from a family background that is more supportive of schooling. Consequently, this selection bias should have been discussed in greater depth: (i) one way to address this would be to adjust the sample using weights based on specific characteristics of the entire population of the County to ensure the representativity of the findings; (ii) otherwise, the limitation must be discussed in the methodology section, clarifying that the study involves a specific population within Troms County and that the results cannot be generalized to the county as a whole.

**2) Statistical analyses:**

The key advantage of using longitudinal data is leveraging time, a key variable to consider when modeling the likelihood of the outcome variable. The authors fitted a discrete-time survival mixture model to identify three (3) latent trajectories of completion status. These latent variables are then used as dependent variable in multinomial logistic models. Apart of this objective of highlighting latent variables and identifying predictors of the different school completion groups, it is worth illustrating “survival” or “failure” table of the 1,676 enrolled in the study in 2010. A good number of summary statistics from the use the discrete time analysis is insightful for the education authorities and for sound education policies: e.g., the classes or years after upper secondary school beginning where there are mor risks of dropouts and cumulative risks of dropouts according to different individual and family background information.
