# Supplementary material for: Completing upper secondary school: Results from a ten-year longitudinal study in Norway
Source: PLoS One. 2026 Nov 6;21(11):e0361808. doi: 10.1371/journal.pone.0361808 (PMC13742913; doi:10.1371/journal.pone.0361808)
Supplement: Supplementary file 5 [file pone.0361808.s006.docx]

*Response to editor’s comments*

*Thank you for the opportunity to further revise the manuscript and submit it again. We hope we have been able to address these comments adequately.*

1. Clarify the relationship between literacy difficulties, GPA, and educational completion
   The revised manuscript acknowledges the association between literacy difficulties, academic performance (GPA), and upper secondary school completion. However, the discussion could be strengthened by providing a more explicit conceptual interpretation of how these factors may be related. A formal mediation analysis is not required, but readers would benefit from a clearer discussion of whether literacy difficulties may influence completion directly, indirectly through academic achievement, or through both pathways.

   *We have added a paragraph to the Discussion, while at the same time trying to refrain from speculating too much beyond our own data.*
2. Improve transparency regarding reproducibility
   The manuscript now provides access to the study dataset through an appropriate repository. However, it remains unclear whether the analytical code, syntax files, or scripts used to generate the latent trajectory models and regression analyses are available. Please clarify whether these materials can be shared and, if so, provide access information. If they cannot be shared, please explicitly state this limitation.

*We have now also uploaded syntax files for the latent trajectory model and regression analyses in dataverse.no. We are awaiting review, but expect the updated version to be published quite fast.*

3. Further clarify the substantive interpretation of the latent classes
   The revised text provides a stronger justification for the latent trajectory approach. Nevertheless, it would be helpful to include a brief statement in the Discussion acknowledging the extent to which the identified latent classes may reflect differences in timing of educational completion rather than qualitatively distinct developmental pathways. This clarification will help readers better understand the substantive interpretation and limitations of the identified trajectories.

*We have revised the Discussion and hope we have managed to adequately	clarified this issue.*
