# Supplementary material for: Completing upper secondary school: Results from a ten-year longitudinal study in Norway
Source: PLoS One. 2026 Nov 6;21(11):e0361808. doi: 10.1371/journal.pone.0361808 (PMC13742913; doi:10.1371/journal.pone.0361808)
Supplement: Peer review history

# Peer review: Decision Letter 0

**DOI:** 10.1371/journal.pone.0361808.r001

**Authors:** Manuel Salas-Velasco

6 Aug 2026

Completing upper secondary school: Results from a ten-year longitudinal study in Norway

PLOS One

Dear Dr. Sæle,

Thank you for submitting your manuscript to PLOS ONE. After careful consideration, we feel that it has merit but does not fully meet PLOS ONE’s publication criteria as it currently stands. Therefore, we invite you to submit a revised version of the manuscript that addresses the points raised during the review process.

In particular, we encourage you to strengthen the methodological justification of the analytical strategy and clarify the interpretation of the identified completion trajectories.

Please provide a detailed point-by-point response to all reviewer comments. In addition, we ask that you address the following editorial concerns (see major issues below).

Please submit your revised manuscript by Sep 20 2026 11:59PM. If you will need more time than this to complete your revisions, please reply to this message or contact the journal office at plosone@plos.org. When you're ready to submit your revision, log on to https://www.editorialmanager.com/pone/ and select the 'Submissions Needing Revision' folder to locate your manuscript file.

- A letter that responds to each point raised by the academic editor and reviewer(s). You should upload this letter as a separate file labeled 'Response to Reviewers'.
- A marked-up copy of your manuscript that highlights changes made to the original version. You should upload this as a separate file labeled 'Revised Manuscript with Track Changes'.
- An unmarked version of your revised paper without tracked changes. You should upload this as a separate file labeled 'Manuscript'.

If you would like to make changes to your financial disclosure, please include your updated statement in your cover letter. Guidelines for resubmitting your figure files are available below the reviewer comments at the end of this letter.

If applicable, we recommend that you deposit your laboratory protocols in protocols.io to enhance the reproducibility of your results. Protocols.io assigns your protocol its own identifier (DOI) so that it can be cited independently in the future. For instructions see: https://journals.plos.org/plosone/s/submission-guidelines#loc-laboratory-protocols. Additionally, PLOS ONE offers an option for publishing peer-reviewed Lab Protocol articles, which describe protocols hosted on protocols.io. Read more information on sharing protocols at https://plos.org/protocols?utm_medium=editorial-email&utm_source=authorletters&utm_campaign=protocols.

As the corresponding author, your ORCID iD is verified in the submission system and will appear in the published article. PLOS supports the use of ORCID, and we encourage all coauthors to register for an ORCID iD and use it as well. Please encourage your coauthors to verify their ORCID iD within the submission system before final acceptance, as unverified ORCID iDs will not appear in the published article. *Only*  the individual author can complete the verification step; PLOS staff *cannot*  verify ORCID iDs on behalf of authors.

We look forward to receiving your revised manuscript.

Kind regards,

Dr. Manuel Salas-Velasco, PhD

Academic Editor

PLOS One

Journal Requirements:

When submitting your revision, we need you to address these additional requirements.

1.Please ensure that your manuscript meets PLOS ONE's style requirements, including those for file naming. The PLOS ONE style templates can be found at

https://journals.plos.org/plosone/s/file?id=wjVg/PLOSOne_formatting_sample_main_body.pdf and

https://journals.plos.org/plosone/s/file?id=ba62/PLOSOne_formatting_sample_title_authors_affiliations.pdf

2. We note that the grant information you provided in the ‘Funding Information’ and ‘Financial Disclosure’ sections do not match.

When you resubmit, please ensure that you provide the correct grant numbers for the awards you received for your study in the ‘Funding Information’ section.

3. Please note that your Data Availability Statement is currently missing the repository name and/or the DOI/accession number of each dataset OR a direct link to access each database. If your manuscript is accepted for publication, you will be asked to provide these details on a very short timeline. We therefore suggest that you provide this information now, though we will not hold up the peer review process if you are unable.

4. Thank you for stating the following financial disclosure:

“The study was funded by Regional Differensiert Arbeidsgiveravgift (RDA) [regional differentiated social security contribution] of Troms County, the Sparebank 1 Nord-Norge foundation (https://snnstiftelsen.no), and UiT the Arctic University of Norway. All funding received by TS. None of the funders played any role in the study design, data collection and analysis, decision to publish, or preparation of the manuscript.”

We note that one or more of the authors is affiliated with the funding organization, indicating the funder may have had some role in the design, data collection, analysis or preparation of your manuscript for publication; in other words, the funder played an indirect role through the participation of the co-authors. If the funding organization did not play a role in the study design, data collection and analysis, decision to publish, or preparation of the manuscript and only provided financial support in the form of authors' salaries and/or research materials, please do the following:

1. Review your statements relating to the author contributions, and ensure you have specifically and accurately indicated the role(s) that these authors had in your study. These amendments should be made in the online form.

2. Confirm in your cover letter that you agree with the following statement, and we will change the online submission form on your behalf:

“The funder provided support in the form of salaries for authors [insert relevant initials], but did not have any additional role in the study design, data collection and analysis, decision to publish, or preparation of the manuscript. The specific roles of these authors are articulated in the ‘author contributions’ section.

5. Please include captions for your Supporting Information files at the end of your manuscript, and update any in-text citations to match accordingly. Please see our Supporting Information guidelines for more information: http://journals.plos.org/plosone/s/supporting-information.

6. If the reviewer comments include a recommendation to cite specific previously published works, please review and evaluate these publications to determine whether they are relevant and should be cited. There is no requirement to cite these works unless the editor has indicated otherwise.

**Additional Editor Comments:**

MAJOR ISSUES

1. Justification of the latent trajectory approach

The central contribution of the manuscript rests on the identification of three latent trajectories: normal progression, delayed completion, and non-completion. However, the manuscript does not sufficiently demonstrate that these classes represent substantively distinct educational trajectories rather than a statistical discretization of a continuous distribution of completion times.

The distinction between “normal progression” and “delayed completion” appears to be primarily temporal. As currently presented, it remains unclear whether the latent class solution is uncovering genuinely distinct populations of students or merely partitioning variation in the timing of graduation.

The revised manuscript should therefore:

- Provide a stronger conceptual rationale for the use of latent classes.

- Clarify how the normal and delayed completion groups differ in terms of actual completion timing.

- Present descriptive information on the distribution of completion times within each latent class.

- Discuss why the latent trajectory approach is preferable to a more direct classification based on observed completion timing.

- Explain what substantive insight is gained from the latent class solution that could not be obtained from observed time-to-completion data.

2. Use of longitudinal information and survival modelling

The manuscript is presented as a ten-year longitudinal study and employs a discrete-time survival mixture model. However, most substantive conclusions are ultimately derived from multinomial logistic regressions predicting latent class membership.

As a result, the temporal dimension of the data appears underutilized. The manuscript would benefit from a more explicit justification of this analytical strategy.

Specifically, the authors should:

- Clarify why multinomial logistic regression was selected as the primary inferential framework following trajectory identification.

- Explain how the chosen approach compares conceptually and analytically with alternative event-history approaches.

- Discuss why the observed completion process was not analysed more directly using event-history or competing-risk frameworks based on educational outcomes over time.

- Consider adding descriptive survival information (e.g., annual completion and non-completion patterns, cumulative completion probabilities, or related summaries) to help readers understand the temporal dynamics underlying the trajectory solution.

I am not requiring a complete reanalysis of the data. However, a substantially stronger justification of the methodological choices is necessary.

3. Educational pathways and interpretation of completion trajectories

The manuscript includes students from both general and vocational tracks. It is not sufficiently clear whether differences in timing of completion may partly reflect structural differences between educational pathways rather than distinct latent student trajectories.

The authors should therefore:

- Provide a clearer description of the Norwegian upper secondary education system for an international readership.

- Explain the duration, objectives, and qualifications associated with the general and vocational tracks.

- Discuss whether differences in institutional organisation, programme structure, or expected completion patterns across tracks may influence the latent trajectory solution.

- Clarify the extent to which the delayed completion group may reflect educational pathway characteristics rather than individual-level heterogeneity.

4. Selection bias and external validity

The manuscript acknowledges that completion rates in the study sample were higher than those observed for the corresponding county population.

This issue deserves a more thorough treatment.

Please:

- Expand the discussion of possible selection effects.

- Clarify the extent to which the study sample can be considered representative of the wider cohort.

- Discuss implications for external validity and generalisability.

- Explain whether weighting procedures were considered and, if not, why they were not feasible.

5. Attrition and follow-up participation

Although the principal outcome was obtained through registry data, the study experienced substantial attrition across questionnaire waves.

The revised manuscript should include:

- An attrition analysis comparing participants who remained in later waves with those who did not.

- A discussion of whether attrition may have affected the interpretation of the broader longitudinal project.

- Clarification of why follow-up questionnaire data were not incorporated into the present analyses.

ADDITIONAL ISSUES

6. Interpretation of GPA and literacy problems

The manuscript shows that GPA dominates the multivariable models and that literacy problems lose statistical significance after GPA is introduced.

Please discuss more explicitly:

- Whether GPA may mediate the relationship between literacy problems and educational completion.

- Whether literacy difficulties contribute indirectly to completion outcomes through academic achievement.

- The implications of this interpretation for educational interventions.

7. Classification of “trait-like” and “state-like” factors

The rationale for classifying literacy problems as a trait-like factor requires clarification.

Please provide a stronger conceptual justification for this classification and discuss whether literacy-related difficulties may be amenable to educational intervention and change over time.

8. Measures

Please provide the number of items included in the literacy problems scale and ensure that all scales are described with sufficient detail to allow replication.

9. Accessibility for an international audience

The paper would benefit from additional contextual information regarding the Norwegian educational system, particularly for readers unfamiliar with the distinction between upper secondary, vocational

10. Data availability and reproducibility

The current Data Availability Statement indicates that the minimal dataset will be deposited in DataverseNO and that a DOI will be provided. However, it remains unclear whether the dataset and associated documentation will be sufficiently available to allow independent verification and replication of the analyses.

Please:

- Update the Data Availability Statement in accordance with PLOS ONE requirements.

- Provide the repository DOI if already available, or clearly explain its status and expected availability upon publication.

- Specify which variables and data files will be made available.

- Clarify whether the data underlying all analyses reported in the manuscript will be accessible without restriction, and if any legal or ethical constraints apply.

- Provide information on the availability of any analysis code, syntax files, or scripts used for data management and statistical analyses, as this would substantially enhance reproducibility.

[Note: HTML markup is below. Please do not edit.]

Reviewers' comments:

Reviewer's Responses to Questions

**Comments to the Author**

1. Is the manuscript technically sound, and do the data support the conclusions?

Reviewer #1: Partly

Reviewer #2: Yes

**********

2. Has the statistical analysis been performed appropriately and rigorously?

Reviewer #1: Yes

Reviewer #2: Yes

**********

3. Have the authors made all data underlying the findings in their manuscript fully available?

The PLOS Data policy

Reviewer #1: No

Reviewer #2: Yes

**********

4. Is the manuscript presented in an intelligible fashion and written in standard English?

Reviewer #1: Yes

Reviewer #2: Yes

**********

Reviewer #1: Beyond the low response rate, the data reveal a selection bias. The study population thus appears more inclined towards schooling and likely comes from a family background that is more supportive of schooling. One way to address this would be to adjust the sample using weights based on specific characteristics of the entire population of the County to ensure the representativity of the findings. Otherwise, the limitation must be discussed in the methodology section, clarifying that the study involves a specific population within Troms County and that the results cannot be generalized to the county as a whole.

It is worth illustrating “survival” or “failure” table of the 1,676 enrolled in the study in 2010. A good number of summary statistics from the use the discrete time analysis is insightful for the education authorities and for sound education policies: e.g., the classes or years after upper secondary school beginning where there are mor risks of dropouts and cumulative risks of dropouts according to different individual and family background information.

Reviewer #2: I’ll start with a minor point: it would be helpful if the section on the questions used also stated how many items are in the ‘Literacy problems’ scale.

As a non-Norwegian, I find it somewhat difficult to grasp the difference between the general and vocational tracks. Another point that might be helpful for readers unfamiliar with the Norwegian school system is: what does the upper secondary school curriculum cover, and for which professions does it provide qualifications?

The article compares the data from the three distinct main groups with one another. This is legitimate and is likely to be of interest to most readers, particularly as the study’s design does not allow for an analysis of why someone belongs to the ‘Completion’ group or the ‘Delayed completion’ group. Whilst the statistical analysis suggests that this was primarily due to the previously achieved GPA, it does not explain why the other criteria surveyed play virtually no role.

However, the strong negative correlation between GPA and literacy problems does leave room for speculation here.

Unfortunately, there is no attrition analysis, which might have provided some insight into this. After all, data is available for most study participants at the first measurement point. So one could (at least in a separate analysis) investigate whether those who completed the questionnaires at two or more measurement points differ from those who only completed the initial questionnaire.

One final suggestion for the authors: in the discussion on page 17, you classify literacy problems as trait-like factors – why is that? If literacy problems were defined as state-like, the obvious question would be: are there elements of upper secondary education designed to bring about a change in this state? And then the question would arise as to the extent to which the levels of the trait-like factors in the three main groups have changed over time.

**********

what does this mean?). If published, this will include your full peer review and any attached files.

If you choose “no”, your identity will remain anonymous but your review may still be made public.

**Do you want your identity to be public for this peer review?**  For information about this choice, including consent withdrawal, please see our Privacy Policy

Reviewer #1: No

Reviewer #2: **Yes:**  PD Dr. Volkhard Fischer, Hannover Medical school

**********

[NOTE: If reviewer comments were submitted as an attachment file, they will be attached to this email and accessible via the submission site. Please log into your account, locate the manuscript record, and check for the action link "View Attachments". If this link does not appear, there are no attachment files.]

To ensure your figures meet our technical requirements, please review our figure guidelines: https://journals.plos.org/plosone/s/figures

You may also use PLOS’s free figure tool, NAAS, to help you prepare publication quality figures: https://journals.plos.org/plosone/s/figures#loc-tools-for-figure-preparation.

NAAS will assess whether your figures meet our technical requirements by comparing each figure against our figure specifications.

Attachment Submitted filename: Review of paper_J-F_KOBIANE.docx

# Author comment: Author response to Decision Letter 1

**DOI:** 10.1371/journal.pone.0361808.r002

4 Oct 2026

Responses are uploaded in a separate file.

Attachment Submitted filename: Response to Reviewers.docx

# Editor report: Decision Letter 1

**DOI:** 10.1371/journal.pone.0361808.r003

**Authors:** Manuel Salas-Velasco

7 Oct 2026

Dear Dr. Sæle,

Please submit your revised manuscript by Nov 21 2026 11:59PM. If you will need more time than this to complete your revisions, please reply to this message or contact the journal office at plosone@plos.org. When you're ready to submit your revision, log on to https://www.editorialmanager.com/pone/ and select the 'Submissions Needing Revision' folder to locate your manuscript file.

- A letter that responds to each point raised by the academic editor and reviewer(s). You should upload this letter as a separate file labeled 'Response to Reviewers'.
- A marked-up copy of your manuscript that highlights changes made to the original version. You should upload this as a separate file labeled 'Revised Manuscript with Track Changes'.
- An unmarked version of your revised paper without tracked changes. You should upload this as a separate file labeled 'Manuscript'.

If you would like to make changes to your financial disclosure, please include your updated statement in your cover letter. Guidelines for resubmitting your figure files are available below the reviewer comments at the end of this letter.

If applicable, we recommend that you deposit your laboratory protocols in protocols.io to enhance the reproducibility of your results. Protocols.io assigns your protocol its own identifier (DOI) so that it can be cited independently in the future. For instructions see: https://journals.plos.org/plosone/s/submission-guidelines#loc-laboratory-protocols. Additionally, PLOS One offers an option for publishing peer-reviewed Lab Protocol articles, which describe protocols hosted on protocols.io. Read more information on sharing protocols at https://plos.org/protocols?utm_medium=editorial-email&utm_source=authorletters&utm_campaign=protocols.

As the corresponding author, your ORCID iD is verified in the submission system and will appear in the published article. PLOS supports the use of ORCID, and we encourage all coauthors to register for an ORCID iD and use it as well. Please encourage your coauthors to verify their ORCID iD within the submission system before final acceptance, as unverified ORCID iDs will not appear in the published article. *Only*  the individual author can complete the verification step; PLOS staff *cannot*  verify ORCID iDs on behalf of authors.

We look forward to receiving your revised manuscript.

Kind regards,

Dr. Manuel Salas-Velasco, PhD

Academic Editor

PLOS One

Journal Requirements:

If the reviewer comments include a recommendation to cite specific previously published works, please review and evaluate these publications to determine whether they are relevant and should be cited. There is no requirement to cite these works unless the editor has indicated otherwise.

Please review your reference list to ensure that it is complete and correct. If you have cited papers that have been retracted, please include the rationale for doing so in the manuscript text, or remove these references and replace them with relevant current references. Any changes to the reference list should be mentioned in the rebuttal letter that accompanies your revised manuscript. If you need to cite a retracted article, indicate the article’s retracted status in the References list and also include a citation and full reference for the retraction notice.

**Additional Editor Comments:**

Minor Issues

1. Clarify the relationship between literacy difficulties, GPA, and educational completion

The revised manuscript acknowledges the association between literacy difficulties, academic performance (GPA), and upper secondary school completion. However, the discussion could be strengthened by providing a more explicit conceptual interpretation of how these factors may be related. A formal mediation analysis is not required, but readers would benefit from a clearer discussion of whether literacy difficulties may influence completion directly, indirectly through academic achievement, or through both pathways.

2. Improve transparency regarding reproducibility

The manuscript now provides access to the study dataset through an appropriate repository. However, it remains unclear whether the analytical code, syntax files, or scripts used to generate the latent trajectory models and regression analyses are available. Please clarify whether these materials can be shared and, if so, provide access information. If they cannot be shared, please explicitly state this limitation.

3. Further clarify the substantive interpretation of the latent classes

The revised text provides a stronger justification for the latent trajectory approach. Nevertheless, it would be helpful to include a brief statement in the Discussion acknowledging the extent to which the identified latent classes may reflect differences in timing of educational completion rather than qualitatively distinct developmental pathways. This clarification will help readers better understand the substantive interpretation and limitations of the identified trajectories.

[Note: HTML markup is below. Please do not edit.]

[NOTE: If reviewer comments were submitted as an attachment file, they will be attached to this email and accessible via the submission site. Please log into your account, locate the manuscript record, and check for the action link "View Attachments". If this link does not appear, there are no attachment files.]

To ensure your figures meet our technical requirements, please review our figure guidelines: https://journals.plos.org/plosone/s/figures

You may also use PLOS’s free figure tool, NAAS, to help you prepare publication quality figures: https://journals.plos.org/plosone/s/figures#loc-tools-for-figure-preparation.

NAAS will assess whether your figures meet our technical requirements by comparing each figure against our figure specifications.

# Author comment: Author response to Decision Letter 2

**DOI:** 10.1371/journal.pone.0361808.r004

13 Oct 2026

Uploaded as a separate file.

Attachment Submitted filename: Response letter.docx

# Editor report: Decision Letter 2

**DOI:** 10.1371/journal.pone.0361808.r005

**Authors:** Manuel Salas-Velasco

16 Oct 2026

Completing upper secondary school: Results from a ten-year longitudinal study in Norway

PONE-D-26-28998R2

Dear Dr. Sæle,

We’re pleased to inform you that your manuscript has been judged scientifically suitable for publication and will be formally accepted for publication once it meets all outstanding technical requirements.

Within one week, you’ll receive an e-mail detailing the required amendments. When these have been addressed, you’ll receive a formal acceptance letter and your manuscript will be scheduled for publication.

An invoice will be generated when your article is formally accepted. Please note, if your institution has a publishing partnership with PLOS and your article meets the relevant criteria, all or part of your publication costs will be covered. Please make sure your user information is up-to-date by logging into Editorial Manager at Editorial Manager® and clicking the ‘Update My Information' link at the top of the page. For questions related to billing, please contact billing support.

If your institution or institutions have a press office, please notify them about your upcoming paper to help maximize its impact. If they’ll be preparing press materials, please inform our press team as soon as possible -- no later than 48 hours after receiving the formal acceptance. Your manuscript will remain under strict press embargo until 2 pm Eastern Time on the date of publication. For more information, please contact onepress@plos.org.

Congratulations on the acceptance of your manuscript, and thank you for submitting your work to the journal.

Kind regards,

Dr. Manuel Salas-Velasco, PhD

Academic Editor

PLOS One

**Additional Editor Comments:**

Please note, however, that during the proof stage you should carefully review the manuscript for any remaining typographical or formatting issues, particularly:

• Verification of the software version reported in the Methods section (e.g., ensuring that the Mplus version is correctly stated and that no track-changes artifacts remain).

• Removal of any residual editing or formatting inconsistencies that may have been introduced during the revision process.

• Confirmation that the data and analysis syntax files referenced in the Data Availability Statement are fully accessible through the cited repository and that the wording of the statement accurately reflects the materials provided.

These are minor points and do not affect the editorial decision. They can be addressed during proof correction prior to publication.

# Editor report: Acceptance letter

**DOI:** 10.1371/journal.pone.0361808.r006

**Authors:** Manuel Salas-Velasco

PONE-D-26-28998R2

PLOS One

Dear Dr. Sæle,

I'm pleased to inform you that your manuscript has been deemed suitable for publication in PLOS One. Congratulations! Your manuscript is now being handed over to our production team.

At this stage, our production department will prepare your paper for publication. This includes ensuring the following:

* All references, tables, and figures are properly cited

* All relevant supporting information is included in the manuscript submission,

* There are no issues that prevent the paper from being properly typeset

You will receive further instructions from the production team, including instructions on how to review your proof when it is ready. Please keep in mind that we are working through a large volume of accepted articles, so please give us a few days to review your paper and let you know the next and final steps.

Lastly, if your institution or institutions have a press office, please let them know about your upcoming paper now to help maximize its impact. If they'll be preparing press materials, please inform our press team within the next 48 hours. Your manuscript will remain under strict press embargo until 2 pm Eastern Time on the date of publication. For more information, please contact onepress@plos.org.

You will receive an invoice from PLOS for your publication fee after your manuscript has reached the completed accept phase. If you receive an email requesting payment before acceptance or for any other service, this may be a phishing scheme. Learn how to identify phishing emails and protect your accounts at https://explore.plos.org/phishing.

If we can help with anything else, please email us at customercare@plos.org.

Thank you for submitting your work to PLOS One and supporting open access.

Kind regards,

PLOS One Editorial Office Staff

on behalf of

Dr. Manuel Salas-Velasco

Academic Editor

PLOS One
